# Puerperal Insanity

**Published:** 1899-12-30

**Authors:** F. St. John Bullen


### Hospital Clinics and Medical Progress.

### PUERPERAL INSANITY.

By F. St. John Bullen, M.R.C.S.

(*Continued from page* 192.)

It is questionable whether any distinction can be drawn between acute delirious mania occurring at the puerperium and at other periods; in each instance it is probably due to some toxæmic condition. In neither case need there be any rise of temperature over that met with in mental disorders; if there be pyrexia over 100 deg. or 101 deg., some complication must certainly be suspected. The most usual dates of onset in puerperal fever and puerperal insanity do not commonly coincide. The former is generally earlier, rarely later than the fourth or fifth day; the latter not often before the fourth or fifth. A variable delay might be expected before the development of mental symptoms, according to the vigour of septic invasion and resistance of nervous system; in some cases delirium may only occur with meningeal complications. There is, in short, a considerable variety in the possible conditions accounting for puerperal insanity and delirium, a complexity largely due to the influence of hereditary predisposition.

These conditions may be summarised thus, in each presupposing heredity:—

Puerperal Insanity: (1) Stress of normal or complicated labour (delay, dystocia, hæmorrhage); (2) some endogenetic toxæmia, the result of shock, constipation, &c.; (3) febrile disturbance due to inflammatory troubles in or around uterus, mammary abscess, &c.; (4) septicæmia, the invasion being only sufficient to disequilibrate, not to seriously reduce, the mind.

Puerperal Delirious Mania: The influence of severe shock, exhaustion, inflammatory disturbance, and even moderate septic invasion on a highly unstable brain.

Grave Puerperal Fever, not necessarily with Heredity: In this last the symptoms of septicæmia, especially the temperature, will indicate the primary importance of the bodily condition.

These cases of septic puerperal mania have undoubtedly been diminished in number by the greater care of the woman both before and after childbirth; they form the main proportion of the, fortunately few, fatal cases (which are about 8 per cent of the whole). That this psychosis is unusually favourable for recovery is to be gathered from the fact that 75 to 80 per cent. of the cases sent to asylums recover; and of the recoveries at least three-quarters are well by six months, and one-half by three months. The acute cases have a really more favourable outlook than the mild ones, in these last treatment often having been delayed. The melancholic cases recover nearly as well as the maniacal, but less rapidly; thus the third month shows the highest ratio of recoveries in mania, and the sixth month in melancholia. The slowest recoveries are in those cases where stupor occurs; a year or more may elapse. Recovery should not be despaired of until at least two years have passed, unless symptoms of organic brain mischief appear. In making the prognosis in puerperal insanity the following points may be kept in mind; most of them have been already discussed at length: (1) Age, date of onset, treatment. Imperfect recoveries or fatal issues are much more common in patients over 30, and in whom treatment has been delayed; recoveries are more tardy in cases where the onset is insidious and postponed; (2) the more numerous the previous attacks, whether of puerperal or other insanity, the more unfavourable the prospect of complete recovery; (3) the stronger the hereditary taint the worse the ultimate outlook, though by no means necessarily is recovery for the time prejudiced; (4) acute delirious cases are very grave, and when marked septic invasion coexists the prospect is as bad as may be; (5) bodily condition (apart from septicæmia, visceral organic disease) affects the progress of recovery more than its eventuality. Fatness occurring disproportionately to mental improvement must not be counted as favourable, neither must the return of the catameina whilst mental disorder is yet marked, and bodily condition still indifferent.

**Treatment of Puerperal Fever.**—Only general principles for guidance can here be stated. The child must, of course, be separated from the mother, and the mother must be guarded night and day by skilled attendants against the dangers of impulsive tendencies so commonly present, and every precaution must be taken in the preparation of the patient's room, and in the removal of all harmful objects. Insomnia, often the earliest symptom, must be overcome by sedatives; choral hydrate, paraldehyde, and bromidia may be tried, but the use of any of these should be discontinued as soon as possible. The frequent anorexia must be met by enforced feeding, which should be resorted to without delay if sufficient nourishment be not taken. During the 24 hours when the case is at all acute, from four to six eggs, three pints of milk with cream as custards, strong soup, &c., should be given; all predigested, if necessary. Stimulants may be freely allowed. If forced alimentation be not followed by great exhaustion it is better to feed four times a day than three; if food is naturally taken it should be administered every three or four hours. In some cases rectal feeding may well supplement oral. At the earliest date allowed by the bodily condition the patient should be sent into the open air, and made to take exercise. It is most important to procure free evacuation of the bowels daily, and to correct the frequently foul condition of the alimentary tract. A mixture containing tr. nuc. vom., tr. bellad., quinine and mist. creosoti B.P. will be found most useful, and can be given with each feed; the dose of quinine can be adjusted to the pyrexia, and extr. cascara sagrada can be added as required. If there is anæmia and no contra-indication exists in the condition of digestive or uterine organs, iron, with or without arsenic, should be ordered, and in the most assimilable form. The treatment of local septic trouble must be based on general principles. Towards the termination of the case progress will sometimes be tardy, a stage of apathy setting in. Change of air and scene under suitable precautions will often act as a stimulant, and hasten recovery.

